# New simplified design methods for engineering barriers around contaminated sites with Cauchy boundaries

**DOI:** 10.1038/s41598-024-59119-y

**Published:** 2024-04-10

**Authors:** Liyilan Zhang, Yiwen Qi, Yuxin Yuan, Yaokai Tan, Guannian Chen, Yan Wang, Tao Wu

**Affiliations:** 1 School of Civil & Environmental Engineering and GeographyScience, Ningbo University, Ningbo, 315211 China; 2 College of Chemistry and Materials Science, Fujian Normal University, Fuzhou, 350005 China; 3 MOE Key Laboratory of Soft Soils and Geoenvironmental Engineering, College of Civil Engineering and Architecture, Zhejiang University, Hangzhou, 310058 China

**Keywords:** Cutoff walls, Cauchy boundary, Breakthrough time, Wall thickness design, Advection‒dispersion equation, Pollution remediation, Civil engineering

## Abstract

Since the 1980s, low-permeability slurry trench cutoff walls have been widely constructed as barriers to retard the migration of contaminants. The thickness of the cutoff walls is a key determinant of the wall service life. Through a series of theoretical derivations, simplified methods for determining the flux limit and concentration limit were proposed to determine the thickness of cutoff walls for contaminated sites with constant pollutant flux. The relative errors of both the flux-based and concentration-based methods increase as the breakthrough criterion of the ratio between the specified limit concentration of the contaminant to the source concentration (C^*^) and the ratio of the limited value of contaminant flux to the constant source flux (F^*^) increases, with a given Peclet number *P*_L_. The maximum relative error reaches 4% and 6% when C^*^ and F^*^ are both 0.1, which covers most practical situations in cutoff wall design. Good agreements of wall thickness were obtained between the proposed simplified methods and analytical solutions via a clear example. The proposed method can efficiently simplify the design process of cutoff walls with high accuracy, providing a basis for containing contaminated sites.

## Introduction

In recent decades, soil pollution has increased due to rapid urbanization and industrialization^1^. With an increasing number of contaminated sites worldwide, slurry trench cutoff walls (hereafter referred to as cutoff walls) have been widely constructed since the 1980s^2^ to retard the migration of contaminants in environmental treatment projects, including subsurface point source pollution^3–5^, soil vapor extraction systems, pump-and-treat methods^6^ and photocatalyst treatments^7,8^. These cutoff walls are usually backfilled with clayey materials such as soil-bentonite or cement-bentonite to achieve a relatively low permeability, which is always controlled to be less than 1 × 10^−9^ m/s^9^. Contaminant transport through cutoff walls can be regarded as a one-dimensional advective–dispersive–adsorptive process, which is usually described by the advection‒dispersion equation, also known as the ADE model^10–12^. More complex models exist in recent years; for example, the Brinkman–Darcy–Bénard model was used to explore natural convection inside an impermeable porous channel with imposed isoflux thermal constraints^13^. The ADE model is still the most commonly used model for the design of engineering barriers invaded by leachate^14–18^. As the migration of contaminants differs greatly from that of pore water, the wall thickness is typically determined via contaminant transport analysis rather than seepage analysis. One key design requirement for cutoff walls is that the breakthrough time of the walls, which is the time corresponding to the contaminant breakthrough of the wall in terms of a specified criterion, should be longer than the designed service life.

Analytical solutions of the ADE model under various boundary condition combinations have been proposed^19–21^ to accurately calculate the process of contaminant migration in cutoff walls. However, the available analytical solutions are nonelementary, as complementary error functions or eigenequations can be seen in their expressions. Thus, the application of these analytical solutions for the design of cutoff walls is nontrivial and generally needs to be determined with a dichotomous method using computers^22^, which has the same challenges as numerical approaches to the ADE model^23–25^. Simplified methods, such as the truncated solution^26^, alternative simplification^27^, advection‒dispersion decoupling model^14^ and fitting simplified equation^15^ based on the analytical solution of Ogata and Banks^28^, were presented to satisfy the design needs of constructions with Dirichlet (first-type, representing a constant concentration boundary) inlet boundaries. As the inlet boundary condition may be more likely to be flux constant according to discussions on the selection of boundary conditions for cutoff walls^29,30^, a design method for cutoff walls with Cauchy (third-type, representing a constant flux boundary) inlet boundary, which has rarely been studied, must be proposed.

New methods for designing cutoff walls with Cauchy boundaries are proposed in this paper. The simplified equations of both methods in terms of the flux criterion and concentration criterion are derived from the simplified method of the Dirichlet inlet boundary. The relative errors between the solutions obtained from the proposed methods and the analytical solutions of the same boundary conditions presented by Lindstrom et al.^31^ are analyzed. Finally, the procedure of using the proposed method in a supposed cutoff wall design is presented in an example.

## Theory

**Figure 1:**
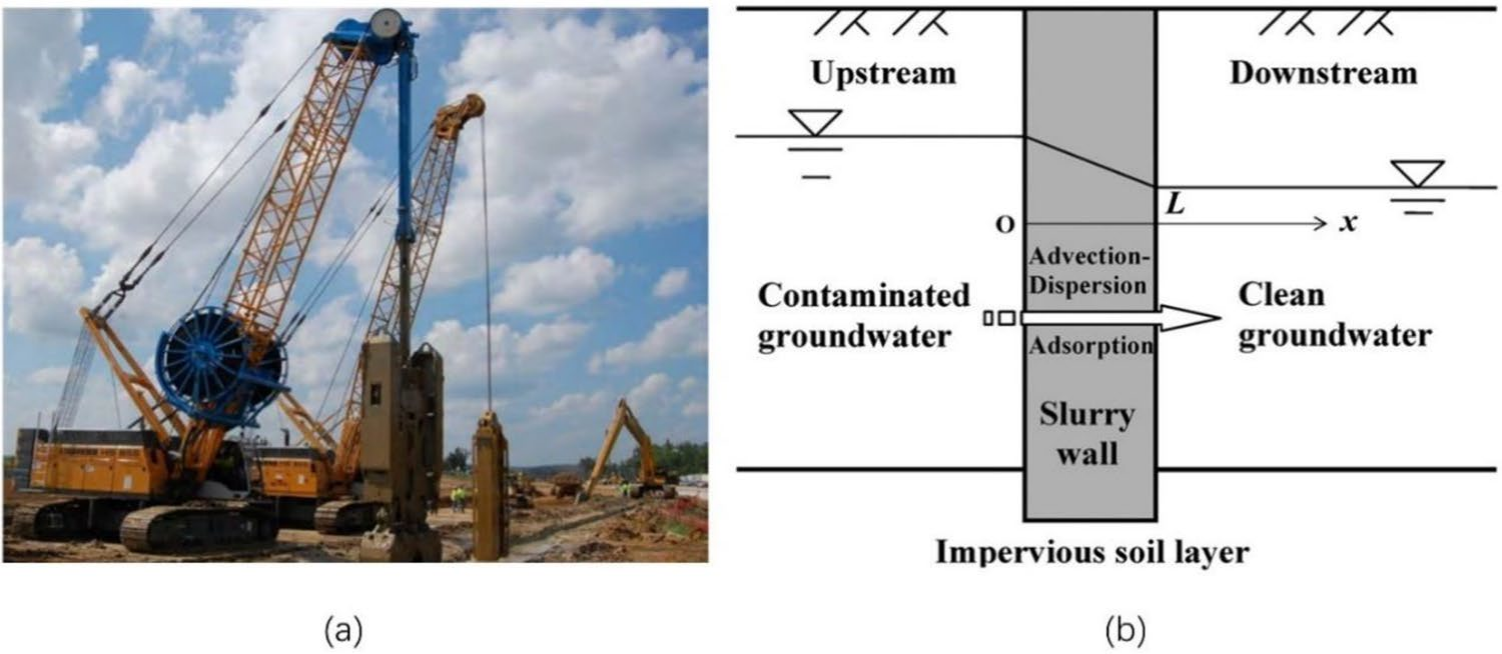
Configuration of a cutoff trench cutoff wall: (**a**) photograph of the excavation process^34^; (**b**) contaminant transport through the wall. In the simulation of actual engineering seepage, a cutoff wall backfilled with homogenous, fully saturated and nondeformable slurry is embedded into the impervious soil layer, and the seepage action of the actual anti-seepage wall is intuitively illustrated through a diagram.

As illustrated in Fig. 1, a cutoff wall backfilled with homogenous, fully saturated and nondeformable slurry embeds into the impervious soil layer. The pore water flow in the field is assumed to be in a steady state with a uniform velocity and direction. The seepage velocity of groundwater is defined as *v*_s_ and is assumed to be perpendicular to the surface of the cutoff walls, which is consistent with the direction of an established one-dimensional coordinate system (*x*). A one-dimensional ADE model^11^ is adopted to describe the contaminant migration in the cutoff walls, and Eq. (1) is obtained^10,32^:

1 $$R_{d} \frac{\partial c}{{\partial t}} = D_{h} \frac{{\partial^{2} c}}{{\partial x^{2} }} - v_{s} \frac{\partial c}{{\partial x}}$$

where *c* is the residual concentration of contaminant in the pore water of backfill, *t* is time, *R*_d_ is the retardation factor of contaminant for the backfill, and *D*_h_ is the hydrodynamic dispersion coefficient of contaminant in the backfill accounting for both the effective molecular diffusion and mechanical dispersion. The first term on the right side of Eq. (1) represents dispersive/diffusive migration of contaminants in the backfill, and the second term represents advective migration. The seepage velocity *v*_s_ can be further written as follows^10^:

2 $$v_{s} = \frac{{v_{d} }}{n} = \frac{kh}{{nL}}$$

where *v*_d_ is the discharge velocity of pore water flow according to Darcy’s law (see Eq. (2)); *n* and *k* are the porosity and hydraulic conductivity of the backfill, respectively; *L* is the thickness of the cutoff wall; and* h* is the water head difference between the two boundaries of the cutoff wall.

The retardation factor *R*_d_ on the left side of Eq. (1), which is a measure of the attenuation capacity of the backfill for the contaminant, represents the effect of adsorption during the migration of the contaminant. For linear, instantaneous and reversible equilibrium adsorption of reactive contaminants, *R*_d_ is a constant that can be given by^14,33^

3 $$R_{d} = 1 + \frac{{\rho_{d} K_{d} }}{n}$$

where *ρ*_d_ is the bulk (dry) density of the backfill and *K*_d_ is the partition coefficient of the contaminant to the backfill.

The contaminant flux, *j*, can be expressed as Ref.^10^:

4 $$j = v_{s} c - D_{h} \frac{\partial c}{{\partial x}}$$

To reduce the number of parameters in the functions, Eq. (1) may be nondimensionalized into the equation below^26^:

5 $$\frac{\partial C}{{\partial T}} = \frac{1}{{P_{L} }}\frac{{\partial^{2} C}}{{\partial X^{2} }} - \frac{\partial C}{{\partial X}}$$

where *C* = *c*/*c*_0_ is the dimensionless pore water concentration of the contaminant, in which *c*_0_ is the source concentration of the contaminant from upstream, *X* = *x*/*L* is the dimensionless distance, *P*_L_ is the column Peclet number^26,35,36^, and *T* is the effective pore volume flow considering the effect of adsorption. The expressions of the dimensionless parameters are:

6-1 $$P_{L} = \frac{{v_{s} L}}{{D_{h} }} = \frac{kh}{{nD_{h} }}$$

6-2 $$T = \frac{{v_{s} t}}{{LR_{d} }} = \frac{kht}{{nR_{d} L^{2} }}$$

The column Peclet number *P*_L_ represents the relative importance of advective migration based on the seepage velocity (*v*_s_) to the dispersive migration based on the hydrodynamic dispersion coefficient (*D*_h_). As noted by Shackelford^26^, the definition of the column Peclet number in Eq. (6-1) should not be confused with that of the Peclet number *P*_e_, in which *L* in Eq. (6-1) is replaced with the size of the soil particle^37^. *h* in Eq. (6-1) is assumed to be a constant related to the most unfavorable scenario of the construction site and is independent of other parameters. For the cutoff wall problem considered, the wall thickness *L* is removed in the further expression of the dimensionless number *P*_L_ (see Eq. (6-1)), which indicates that the value of *P*_L_ is dependent only on the properties of the backfill slurry and hydrogeological environment of the field and can be considered constant for each single cutoff wall design.

The effective pore volume flow *T*, which is proportional and only dependent on *t*, as shown in Eq. (6-2), is a dimensionless time variable that represents the time relative to the breakthrough time of the wall under the advection-adsorption process. The most commonly used definition of dimensionless time, which is also called pore volume flow (PVF), does not include the effect of adsorption^30^ and is not the same as the dimensionless time *T* defined in this paper. As seen in the further expression of *T* (see Eq. (6-2)), the breakthrough time of the cutoff walls is proportional to the retardation factor *R*_d_ and the square of the wall thickness *L*^2^, which fits the conclusion from centrifuge tests conducted by Shu et al.^16^.

The initial contaminant concentration within the cutoff walls is regarded as zero since slurry cutoff walls are generally constructed with uncontaminated backfill. A Cauchy boundary representing constant flux^30^ at the entrance boundary of the cutoff walls, which is chosen as the origin of the coordinate system *x*, is assumed in this paper as follows:

7 $$J(0,T) = \frac{j(0,t)}{{v_{s} c_{0} }} = C(0,T) - \frac{1}{{P_{L} }}\frac{\partial C(0,T)}{{\partial X}} = 1$$

Slurry cutoff walls are mostly constructed in planes, which indicates that contaminants further migrate in porous media after breaking through the cutoff walls. Thus, a semi-infinite outlet boundary, which can represent the scenario in which the downstream soil is relatively homogenous and sufficiently large such that the downstream soil may be regarded as a semi-infinite space, is more accurate for describing the in situ boundary of the cutoff walls:

8 $$\partial C(\infty ,T) = 0$$

The calculation results of the semi-infinite boundary and finite boundary are basically the same in the early stage of breakthrough of the cutoff wall. For finite boundaries, Fourier series may be used^38,39^, resulting a much more complicated derivation process, can be solved in the future researches.

Analytical solutions of Eq. (1) with different types of boundary conditions have been solved previously. The analytical solution of Eq. (1) into boundary conditions Eqs. (7) and (8) gives the following equation^12^:

9 $$\begin{gathered} C(X,T) = \frac{1}{2}{\text{erfc}}\left[ {\frac{1}{2}\left( {X\sqrt {\frac{{P_{L} }}{T}} - \sqrt {P_{L} T} } \right)} \right] \hfill \\ + \sqrt {\frac{{P_{L} T{\prime} }}{\pi }} e^{{ - \frac{{P_{L} (X - T)^{2} }}{4T}}} - \frac{1}{2}(1 + P_{L} + P_{L} T)e^{{XP_{L} }} {\text{erfc}}\left[ {\frac{1}{2}\left( {X\sqrt {\frac{{P_{L} }}{T}} + \sqrt {P_{L} T} } \right)} \right] \hfill \\ \end{gathered}$$

Since the Peclet number *P*_L_ is a constant, the design of the cutoff walls with Cauchy boundaries actually involves solving Eq. (9) for the specified *T*, which fits the upper limit of the breakthrough standard. The breakthrough time for a constructed cutoff wall or required wall thickness that satisfies the expected breakthrough time can then be calculated from Eq. (6-2).

## Design method based on the flux limit

### Methodology

For cutoff walls with constant inlet contaminant flux, controlling the flux downstream of the walls, which is also a metric of the total amount of pollutants discharged from the contaminated sites, could be an ideal starting point for simplified design methods. The ratio of the limited value of contaminant flux (f^*^) to the constant source flux, which is *v*_s_*c*_0_, is defined as the breakthrough criterion of flux F^*^. The breakthrough time *t*_b_ of the cutoff walls is defined as the time when the cutoff walls reach the criterion of breakthrough, that is, the exit flux (in which *X* = 1) reaches the breakthrough criterion of flux F^*^ in this section. Correspondingly, *T*_b_ is a dimensionless form of the breakthrough time *t*_b_.

The expression of the analytical solution for contaminant flux under the Cauchy boundary is similar to that of the residual concentration under the Dirichlet boundary^28^. Based on the simplified solution for the analytical solution of the Dirichlet boundary^14^, the simplified flux equation for the Cauchy inlet problem may be written in a more general form from the analytical solution as follows:

10 $$\begin{gathered} F(X,T) = \frac{f(X,T)}{{v_{s} c_{0} }} \hfill \\ = \frac{1}{2}{\text{erfc}}\left[ {\frac{1}{2}\left( {X\sqrt {\frac{{P_{L} }}{T}} - \sqrt {P_{L} T} } \right)} \right] + \frac{1}{2}\exp (XP_{L} ){\text{erfc}}\left[ {\frac{1}{2}\left( {X\sqrt {\frac{{P_{L} }}{T}} + \sqrt {P_{L} T} } \right)} \right] \hfill \\ \cong {\text{erfc}}\left[ {\frac{1}{2}\left( {X\sqrt {\frac{{P_{L} }}{T}} - \sqrt {P_{L} T} } \right)} \right] \hfill \\ \end{gathered}$$

**Figure 2:**
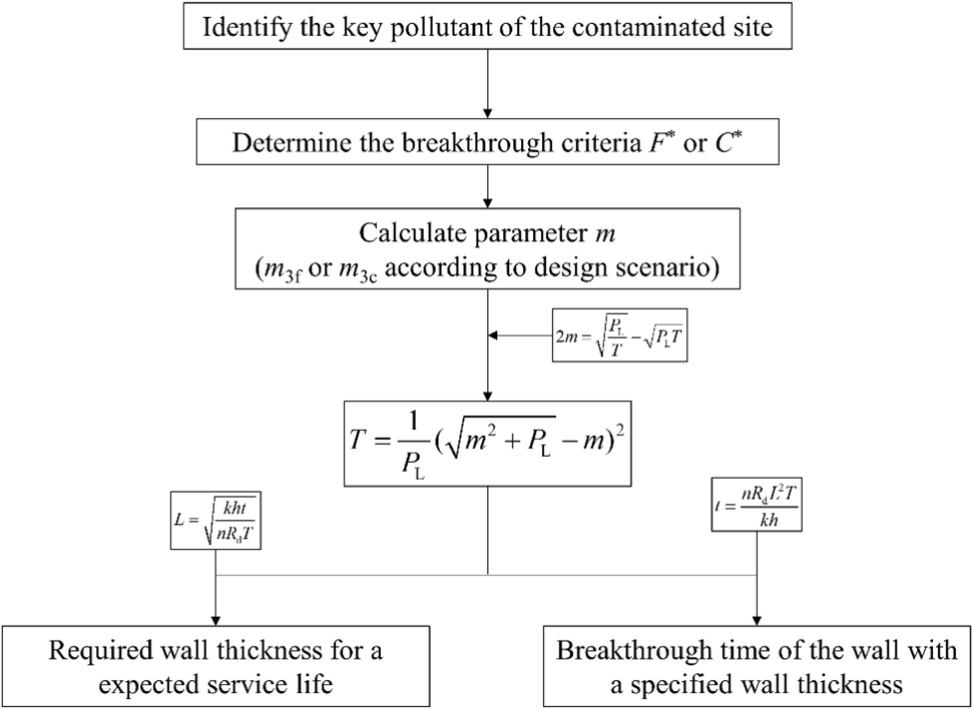
Flow chart for the simplification process of the flux limit method. With the value of *T*_b_ determined, key parameters used in cutoff wall design can be further ascertained.

A flow chart for the simplification process of the method using Eq. (10) is shown in Fig. 2. The F^*^ value of the key pollutant parameter, i.e., heavy metal content for mining sites or chemical oxygen demand (COD) for municipal landfills, is first calculated. The variable *m*_3f._ is then defined as a simple expression for the content in the erfc function of Eq. (10). Therefore, the value of *m*_3f._ equals the solution to the inverse function of the error function complement, that is, erfc^−1^, of F^*^ in this section. For F^*^ values ranging from 0.001 to 0.1, which is a rather large range for common scenarios, the following approximating formula is proposed by the least-square fitting method for the determination of *m*_3f._:

11 $$\left\{ {\begin{array}{*{20}l} {m_{3f} = 3.56 - 3.33F^{{*^{{^{0.142} }} }} } \hfill & {\left( {0.001 < F^{*} < 0.1} \right)} \hfill \\ {m_{3f} = 1.48 - 3.79F^{*} + 5.35F^{{*^{2} }} - 3.55F^{{*^{3} }} } \hfill & {\left( {0.1 < F^{*} < 0.6} \right)} \hfill \\ \end{array} } \right.$$

The relative error of Eq. (11) to the original erfc^−1^ function is less than 1.5% for F* values ranging from 0.001 to 0.6. The expression of Eq. (10) is then transformed into a simple quadratic equation, and the solution of the equation can be explicitly solved as:

12 $$T_{b} = \frac{1}{{P_{L} }}\left( {\sqrt {m_{3f}^{2} + P_{L} } - m_{3f} } \right)^{2}$$

With the value of *T*_b_ determined, the key parameters used in the cutoff wall design can be further ascertained, as illustrated in Fig. 2. The wall thickness needed to satisfy the designed service life of *t*_b_ can be obtained directly by introducing the definition of *T*_b_ (see Eq. (6-2)). For a cutoff wall with a specified thickness *L*, the breakthrough time for a specified F^*^ can also be estimated via the same method.

Equation (12) indicates that the wall thickness is directly determinable in the proposed method. To avoid early breakthrough due to field nonuniformity, a safety factor of 1.1–1.3 is suggested to be multiplied by the wall thickness obtained by the proposed method. Therefore, by simplifying the contaminant transport analysis-based design method, the normal searching process when using an analytical solution is not needed.

### Error analysis

**Figure 3:**
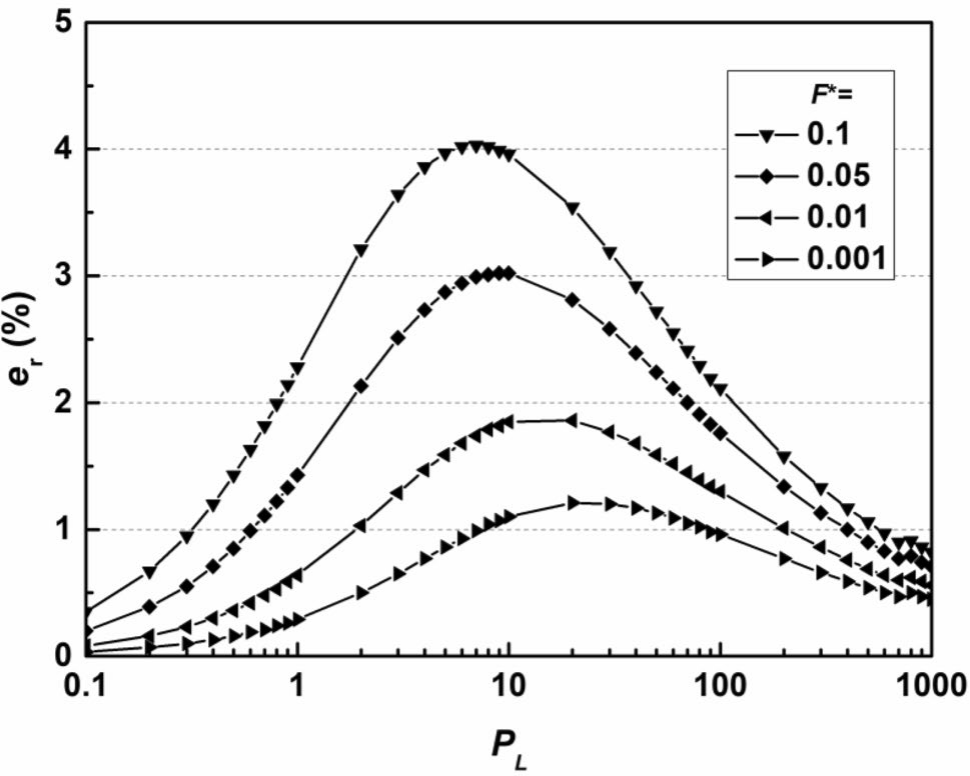
Relationship between the relative error *e*_r_ and *P*_L_ for the flux-based design method.

The relative error, *e*_r_, of the thickness of the wall *L* obtained by the simplified method compared to that obtained by the analytical solution of Eq. (9) is calculated based on the relationship between *L* and *T* as follows:

13 $$e_{r} = \left( {\frac{{L_{{\text{s}}} }}{{L_{a} }} - 1} \right) \times 100\% = \left( {\sqrt {\frac{{T_{a} }}{{T_{s} }}} - 1} \right) \times 100\%$$

where parameters with subscript ‘s’ are the values obtained by the simplified method and parameters with subscript ‘a’ are the analytical solutions of the analytical equations, Eqs. (9, 10), obtained via the Newton–Raphson searching method^40^. The range of *P*_L_ used in the error analysis is chosen to be 0.1 ~ 1000, as the value of hydraulic conductivity of backfills, *k*, is typically controlled within the range of 1 × 10^−11^ m/s to 1 × 10^−9^ m/s^9^, and the hydrodynamic dispersion coefficient, *D*_h_, is in the range of 1 × 10^−10^ m^2^/s to 1 × 10^−9^ m^2^/s^22^ for many cases of cutoff walls. The range of* P*_L_ values of 0.1 to 1000 is sufficient to include all the conditions that may be covered in actual construction scenarios (typically within the range of 1 ~ 20), as shown in Eq. (6-1). The relationship between *e*_r_ and *P*_L_ for different values of F^*^ ranging from 0.001 to 0.1 is shown in Fig. 3.

The value of *e*_r_ is positive for all scenarios, which results from the proposed method being conservative. The relative error is relatively high for higher F^*^ and intermediate *P*_L_ values and reaches a maximum value of approximately 4% for F^*^ equal to 0.1 and *P*_L_ equal to 5 for the calculated scenarios. The proposed method provides sufficient accuracy for design, as the verification range is large enough for common design.

The relative significance of dispersive flux to the whole migration process can be calculated via the ratio *J*_d_/*J*_ss_, in which *J*_d_ and *J*_ss_ are the dispersive flux and the total flux at the end of the cutoff walls, respectively. The values of *J*_d_ and *J*_ss_ are expressed as follows:

14-1 $$J_{d} = - nD_{h} \frac{\partial c}{{\partial x}}$$

14-2 $$J_{ss} = v_{{\text{s}}} c - nD_{h} \frac{\partial c}{{\partial x}}$$

By substituting Eq. (9) into Eqs. (14-1) and (14-2), the value of *J*_d_/*J*_ss_ can be calculated analytically. The ratio of dispersive flux to the total flux at the time of breakthrough can also be written in a relatively concise form with the simplified equation Eq. (10), which is the ratio between the second term and the first term of the analytical solution presented by Ogata and Banks^10^:

15 $$\frac{{J_{d} }}{{J_{ss} }} = \frac{{\exp (P_{L} ){\text{erfc}}\left[ {\frac{1}{2}\left( {\sqrt {\frac{{P_{L} }}{{T_{b} }}} + \sqrt {P_{L} T_{b} } } \right)} \right]}}{{{\text{erfc}}\left[ {\frac{1}{2}\left( {\sqrt {\frac{{P_{L} }}{{T_{b} }}} - \sqrt {P_{L} T_{b} } } \right)} \right]}}$$

**Figure 4:**
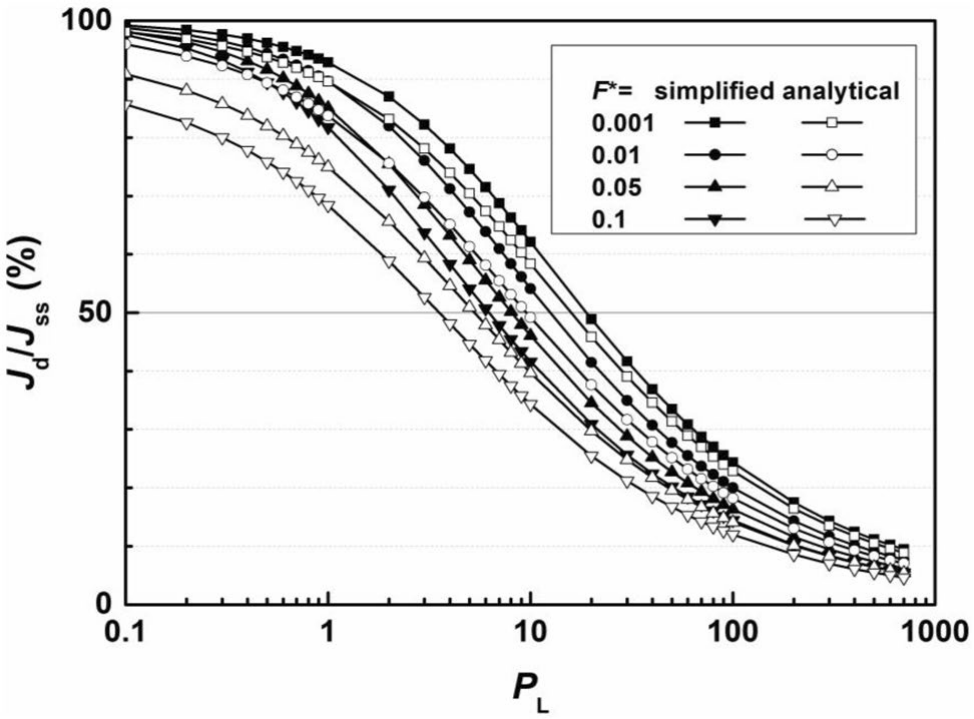
Ratio of dispersion flux to total flux.

The values of *J*_d_/*J*_ss_ are calculated at the breakthrough time for different breakthrough criteria of flux F^*^ both simplistically and analytically. The relationship between *J*_d_/*J*_ss_ and *P*_L_ for various F^*^ values is shown in Fig. 4. According to the analytical results, the values of *J*_d_/*J*_ss_ ranged from 85 to 98% when the *P*_L_ was 0.1, illustrating a dispersion-dominated migration process; then, *J*_d_/*J*_ss_ decreased as the *P*_L_ increased and finally decreased to 4 to 9% as the *P*_L_ increased to 1000, where advection obviously dominated. *J*_d_/*J*_ss_ reaches approximately 50% when the *P*_L_ ranges from 4 to 20, where advection and dispersion have the same degree of impact on the migration process, coinciding with the peak of the error analysis.

Compared to the results from the analytical solutions, the *J*_d_/*J*_ss_ calculated from simplified Eq. (15) is always greater, and the difference between the results from the two methods is mostly less than 10%, proving that the simplified method is more accurate. The difference between the results of the two methods is larger in low F^*^ scenarios, which also proves that the relative error is greater under relatively high F^*^ values.

## Design method based on the concentration limit

### Methodology

A design method based on the flux limit provides total control of the leaked pollutant from the contaminated site. However, in real practice, the values of the contaminant flux from the sites are difficult to determine, and the value of the resident concentration is more commonly used for the evaluation of contaminant controls. Limits to the concentration of contaminants are also more commonly adopted in environmental standards with respect to flux limits^41^. According to Chen et al.^42^, the simplified solution of the flux (Eq. (10)) can be transformed into an equation for the resident concentration. The relative error is less than 5% for most practical cutoff wall projects, which can be applied to continue to simplify the calculations (the error analysis of the equation may be found in the referenced paper):

16 $$C(X,T) = {\text{erfc}}\left[ {\frac{1}{2}\left( {X\sqrt {\frac{{P_{L} }}{T}} - \sqrt {P_{L} T} } \right)} \right] - \exp (XP_{L} ){\text{erfc}}\left[ {\frac{1}{2}\left( {X\sqrt {\frac{{P_{L} }}{T}} + \sqrt {P_{L} T} } \right)} \right]$$

Although it is already much simpler than the analytical solution of Eq. (9), Eq. (16) is still too complex to be directly used in cutoff wall design and needs further simplifications. In the following deductions, the dimensionless length *X* will always equal 1, and the dimensionless concentration will be replaced by breakthrough criterion C^*^ to solve the breakthrough problem of the cutoff walls. Similar to F^*^, which was defined previously, the ratio of the limited value of the specified concentration of the contaminant (c^*^) to the source concentration (*c*_0_), which is defined as the breakdown criterion of concentration C^*^, c^*^, can be obtained from practical projects or regulations.

The simplifying method is proposed by combining the two terms of Eq. (16). Equation (16) can be initially expanded as follows through the definition of the erfc function:

17 $$C(1,T) = \frac{2}{\sqrt \pi }\int_{{\frac{1}{2}\sqrt {\frac{{P_{L} }}{T}} - \frac{1}{2}\sqrt {P_{L} T} }}^{ + \infty } {e^{{ - \eta_{1}^{2} }} d\eta_{1} } - \frac{2}{\sqrt \pi }\int_{{\frac{1}{2}\sqrt {\frac{{P_{L} }}{T}} + \frac{1}{2}\sqrt {P_{L} T} }}^{ + \infty } {e^{{ - \eta_{2}^{2} + P_{L} }} d\eta_{2} }$$

With the transformation of η_3_^2^ = η_2_^2^ − *P*_L_, the two terms of Eq. (17) could have the same integral range and can be combined as:

18 $$C(1,T) = \frac{2}{\sqrt \pi }\int_{{\frac{1}{2}\sqrt {\frac{{P_{L} }}{T}} - \frac{1}{2}\sqrt {P_{L} T} }}^{ + \infty } {\frac{{\sqrt {\eta^{2} + P_{L} } - \eta }}{{\sqrt {\eta^{2} + P_{L} } }}e^{{ - \eta^{2} }} d\eta }$$

The integral in Eq. (18) is actually a modification to the expansion of the erfc function. The correction term of Eq. (18) is simplified with an exponential function within the range of 0 < η < 3, which is the main range that affects the solution of the integral (i.e., the value of the unmodified function, erfc (3), decreases to approximately 2.2 × 10^−5^ and is nearly negligible for most scenarios). Equation (18) can then be derived as:

19 $$C^{*} = \frac{2}{\sqrt \pi }\int_{{\frac{1}{2}\sqrt {\frac{{P_{L} }}{{T_{b} }}} - \frac{1}{2}\sqrt {P_{L} T_{b} } }}^{ + \infty } {\exp \left( { - \frac{1.16\eta }{{\sqrt {P_{L} } }} - \eta^{2} } \right)d\eta } = \exp \left( {\frac{0.34}{{P_{L} }}} \right){\text{erfc}}\left( {m_{3c} + \frac{0.58}{{\sqrt {P_{L} } }}} \right)$$

where *m*_3c_ is a parameter with the same expression as *m*_3f_ for concentration-based design. The process of using the concentration-based design method is the same as that for the flux-based method, as shown in Fig. 2. Based on Eq. (19), the determination of *m*_3c_ is similar to that of *m*_3f_, which is expressed as follows. The dimensionless time *T* can then be calculated by Eq. (12), which is the same as the flux-based design, and further transformed to the required wall thickness or predicted service life.

20 $$\left\{ {\begin{array}{*{20}l} {m_{3c} = 3.56 - 3.33\exp \left( { - \frac{0.048}{{P_{L} }}} \right)C^{{*}{^{0.142} }} - \frac{0.58}{{\sqrt {P_{L} } }}} \hfill & {\left( {0.001 < C^{*} < 0.1} \right)} \hfill \\ \begin{gathered} m_{3c} = 1.48 - 3.79\exp \left( { - \frac{0.34}{{P_{L} }}} \right)C^{*} + 5.35\exp \left( {\frac{0.68}{{P_{L} }}} \right)C^{{*^{2} }} \hfill \\ - 3.55\exp \left( { - \frac{1.02}{{P_{L} }}} \right)C^{{*^{3} }} - \frac{0.58}{{\sqrt {P_{L} } }} \hfill \\ \end{gathered} \hfill & {\left( {0.1 < C^{*} < 0.6} \right)} \hfill \\ \end{array} } \right.$$

### Error analysis

**Figure 5:**
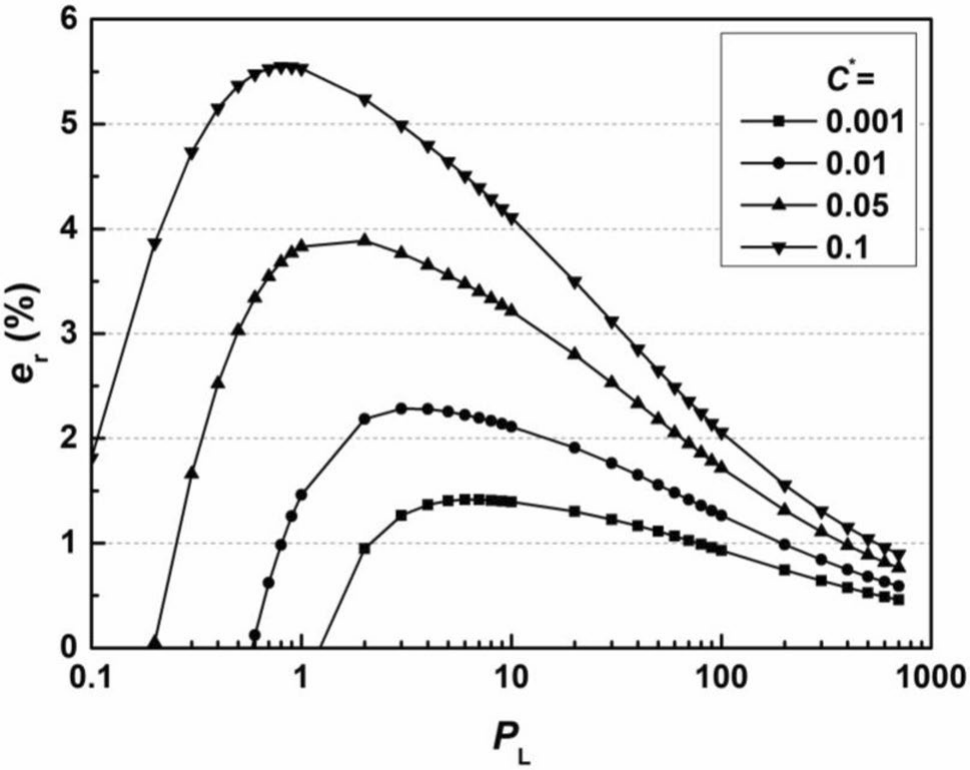
Relationship between the relative error *e*_r_ and *P*_L_ for the general concentration-based design method.

A series of error analyses are also performed for the proposed method for concentration-based design. Similar to the flux-based design, the relative error of the dimensionless time *T* calculated from the simplified solution of Eq. (19) was compared to that of the analytical solution of Eq. (9) using Eq. (13). The relative errors for the proposed method for *P*_L_ values ranging from 0.1 to 1000 and C^*^ values ranging from 0.001 to 0.05 are shown in Fig. 5.

The relative error for Eq. (17) has a similar trend to that of the flux-based design method; that is, *e*_r_ is greater in the high C^*^ and medium *P*_L_ scenarios. The peak relative errors in Fig. 5 are 1.4%, 2.3%, 3.8% and 5.5% when the breakthrough criterion is 0.001, 0.01, 0.05 and 0.1, respectively, which are slightly greater than the relative errors of the flux-based method. The accuracy of the proposed concentration-based method is fully acceptable because the value of *e*_r_ is less than 4% for most scenarios and the peak relative error is less than 6%.

A comparison between the proposed method and that of Chen et al.^42^, which is another simplified and fitted design method for Cauchy boundaries under a dispersion dominating scenario, was also conducted. The calculation equation of the compared model is:

21 $$\sqrt {\frac{{P_{L} }}{T}} = 12.55 - 12\left( {\frac{{C^{*} }}{{P_{L} \exp (P_{L} /2)}}} \right)^{0.044}$$

**Figure 6:**
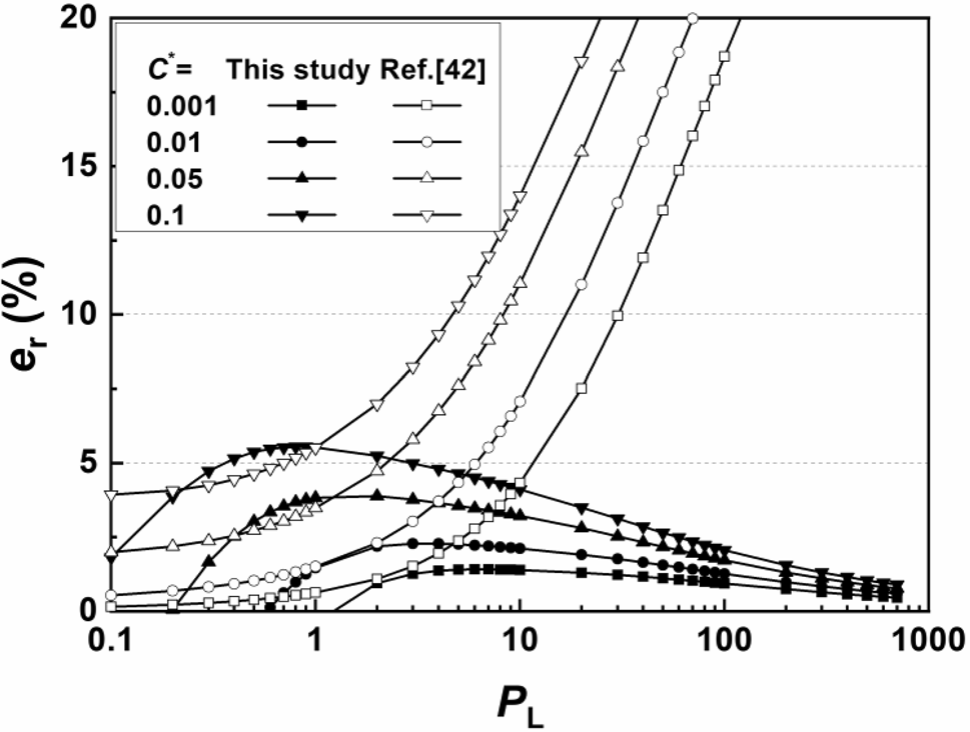
Comparisons between the relative error *e*_r_ for the general concentration-based design method.

Comparisons between the relative errors of the breakthrough time calculated by the proposed method and the simplified equation from Chen et al.^42^ are illustrated in Fig. 6. It can be concluded that the relative errors of the proposed method are nearly always lower than those of the compared models even under dispersion-dominant scenarios since the simplification process of the proposed method applies a modification without many limitations, as shown in Eqs. (17, 18). The previous method performed only slightly better in the minorly distributed range with high C^*^ values, as it is derived in low-*P*_L_ scenarios, conducted with a rough neglection to a term containing *P*_L_. It is verified that the proposed concentration-based method has good performance, providing a closer predicted breakthrough time or needed wall thickness to the analytical solutions. For advection dominating scenarios, the relative errors of the previous method increase unlimitedly and are demonstrated to be unapplicable in such scenarios, while the proposed method still has good performance. The proposed method is applicable for the design of cutoff walls with Cauchy inlet boundaries for all scenarios.

## Cutoff wall example analysis

The procedure for applying the proposed method for determining the wall thickness of cutoff walls is illustrated in this section. For the example considered, a cutoff wall is constructed to retard the migration of per- and polyfluoroalkyl substances (PFAS) from a municipal landfill for 50 years, which is a common service life for an MSW landfill for the degradation of organic pollutants. Soil-bentonite, which is a low-permeability clay barrier material that is typically used in containment systems, compacted to a porosity of 0.4 is used in construction, and the corresponding hydraulic conductivity of the backfill is 1 × 10^–9^ m/s^9^. The water head difference between the contaminated site and groundwater level is assumed to be 1.0 m for a medium flow rate. The retardation factor of the backfill to PFAS is taken as 4.0 based on Wang et al.^43^, and the hydrodynamic dispersion coefficient is set to 5 × 10^–10^ m^2^/s based on Guo et al.^44^. The required wall thickness of the cutoff walls satisfying the two environmental standards of F^*^ = 0.01 and C^*^ = 0.01 can be determined as follows.

The value of *P*_L_ is first determined by Eq. (6-1) and calculated to be 5.0. The value of parameter *m* is then approximated via Eqs. (11) and (20) for the flux-based and concentration-based methods. In the results, *m*_3f._ = 1.83 and *m*_3c_ = 1.60, and the corresponding required wall thicknesses for F^*^ = 0.01 and C^*^ = 0.01 are determined to be 2.10 m and 1.93 m, respectively. A thickness of approximately 2 m is needed to retard the migration of PFAS through cutoff walls, which is greater than the thickness generally used in practical projects, that is, 0.9 to 1.5 m. The poor performance of the cutoff wall is due to its low adsorption of PFAS. The required wall thickness will be halved if *R*_d_ can be advanced to 16, which is 4 times the value used in the current calculation, as previously analyzed in Eq. (6-2). The retardation efficiency of soil-bentonite slurry cutoff walls would be much improved if the symbolic pollutant of the landfill replaced heavy metals, which can be easily adsorbed by clay barrier materials. On the other hand, the wall thickness for the flux-based design is approximately 9% greater than that of the concentration-based design, as the control demand of its outflow concentration is slightly stricter due to the impact of dispersion flux.

**Figure 7:**
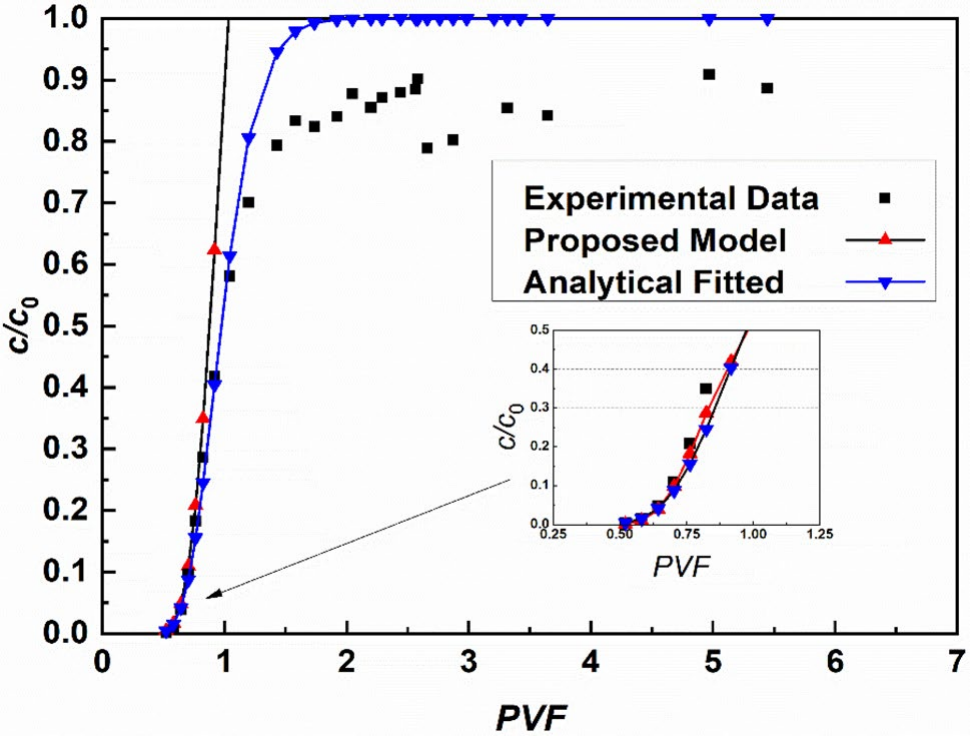
Comparison of the breakthrough curves predicted by the proposed method.

An additional reverse verification for the proposed method is applied to the experimental data of a long-term column test^45^. The Peclet number, *P*_L_, fitted for a 500-ppm column is approximately 34, while the *x*-axis is modified in the effective pore volume (Eq. (6-2)) form with a retardation factor of 13. The breakthrough curve calculated by the proposed method, Eq. (19), is labeled in Fig. 7, along with the experimental data and the curve fitted by the ADE model. It can be illustrated that the proposed equation has good agreement with the tested data, with correlation indices of 0.914 and 0.977 comparing with tested data and analytical results, respectively, when *c*/*c*_0_ value is less than 0.3. It is illustrated that the proposed method provides satisfactory accuracy when used for the design of cutoff walls. However, the breakthrough curve of the proposed method increases nonlinearly as the actual effluent concentration approaches the source concentration, which is due to the simplifications made to the method. Thus, the proposed equations cannot be applied as substitutes for the analytical solutions.

The application of the proposed method can also be extended to more complicated scenarios with the assistance of other models. For example, the dual porosity model, which is always recommended for describing complex non-Fickian contaminant transport in soil, is difficult to calculate. The proposed methods are available for the design of engineering barriers constructed with non-Fickian materials and Cauchy inlet boundaries, with the dual porosity migration process simplified to an ADE process, as performed in Chen et al.^46^.

## Conclusion

Novel simplified methods for determination of the thickness of cutoff walls have been proposed via a series of theoretical derivations to the Cauchy boundary analytical solution, which is practical for contaminated sites with constant pollutant flux seldom considered in existing researches. The relative errors of both the flux-based and concentration-based methods are greater with higher breakthrough criteria (C^*^ and F^*^) for a given Peclet number *P*_L_ but are not greater than 4% and 6% for common practical scenarios in cutoff wall design, where the breakthrough criterion is usually no greater than 0.1. The relative error of the proposed method is greater with intermediate *P*_L_ values, and is mostly lower than that of existing Cauchy boundary simplifying method. Close results of wall thickness were obtained using the proposed simplified methods and analytical solutions via a clear example, a reverse example also verifies high correlation between the proposed method calculated results to the experimental column test data. The proposed method can efficiently simplify the design process of cutoff walls with high accuracy, providing a basis for containing contaminated sites. Finally, careful comparison of the boundary conditions and proper calculations with suitable analytical solutions should be performed before extending the proposed method to other contaminant transport problems, and a safety factor is also suggested to avoid early breakthroughs caused by field nonuniformity.

## Data Availability

The datasets used and/or analyzed during the current study are available from the corresponding author upon reasonable request.

## Abbreviations

*C*^*^: Specified limit concentration of the contaminant to the source concentration
*c*_0_: Source concentration
*c*^***^: Specified breakthrough concentration
*F*^*^: The speicified breakthrough criterion of flux
*P*_L_: Column Peclet number
*v*_s_: Seepage velocity of groundwater
*c*: Resident concentration of contaminant in the pore water of backfill
*t*: Time
*R*_d_: Retardation factor
*D*_h_: Hydrodynamic dispersion coefficient
*j*: Contaminant flux
*v*_d_: Discharge velocity of pore water flow
*n*: Porosity
*k*: Hydraulic conductivity
*L*: Thickness of the cutoff wall
*h*: The water head difference between the two boundaries of the cutoff wall
ρ_d_: Bulk (dry) density of the backfill
*K*_d_: Partition coefficient of contaminant to the backfill
*C*: *c*/*c*_0_, which is the dimensionless pore water concentration of contaminant
*X*: *x*/*L*, which is the dimensionless distance
*T*: Effective pore volume flow considering the effect of adsorption
*f*^*^: Limited value of contaminant flux
*T*_b_: Effective pore volume flow of the breakthrough time *t*_b_
*m*_3f_: Design constant for *F*^***^
*e*_r_: Relative error
*J*_d_: Dispersive flux at the end of the cutoff walls
*J*_ss_: Total flux at the end of the cutoff walls
*m*_3c_: Design constant for *C*^*^
